# Accuracy of Microcomputed Tomography in Detecting Dentinal Cracks: A Correlative Study with Scanning Electron and Operative Microscopy

**DOI:** 10.1155/2021/5571123

**Published:** 2021-05-15

**Authors:** Andrea F. Campello, Marília F. Marceliano-Alves, José C. Provenzano, Simone C. Loyola, José F. Siqueira, André G. Machado, André L. Machado, Ricardo T. Lopes, Maurício M. Paiva, Flávio R. F. Alves

**Affiliations:** ^1^Faculty of Dentistry, Iguaçu University, Nova Iguaçu, RJ, Brazil; ^2^Department of Endodontics, Grande Rio University (UNIGRANRIO), Duque de Caxias, RJ, Brazil; ^3^Department of Nuclear Energy, Rio de Janeiro Federal University, Rio de Janeiro, RJ, Brazil; ^4^National Institute of Technology, Rio de Janeiro, RJ, Brazil

## Abstract

The aim of the study was to evaluate the accuracy of microcomputed tomography (mCT) to detect dentinal cracks when compared with scanning electron microscopy (SEM) and operating microscopy (OM). Different conditions of pixel size (10 or 17 *μ*m), sample moisture (dry/moist), and transillumination (with/without) were evaluated. Additionally, the influence of the dentinal defect width on its detection was analyzed. The root canals of human mandibular incisors were prepared with the Reciproc R40 instrument (VDW, Munich, Germany). The roots were sectioned 5 and 10 mm from the apex, and mCT scans of middle and apical segments were performed at two pixel sizes: 10 *μ*m and 17 *μ*m, under dry and moist conditions (groups: 10dry, 10moist, 17dry, and 17moist). The operating microscope was used with and without transillumination (groups: OMTrans and OM). Findings showed that accuracy was moderate for the 10dry, 10moist, and OMTrans groups, poor for OM and very poor for 17dry and 17moist. The thickness of the dentin crack significantly influenced its detection by mCT using the resolution of 10 *μ*m in both dry and wet conditions (*P* = .002), 17 *μ*m in the dry condition (*P* = .002), and by the operating microscope using transillumination (*P* = .009). Some cracks visualized in SEM were not detected by mCT and an operating microscope. Not only the mCT resolution but also the sample moisture condition and the dentinal crack width can significantly influence its detection.

## 1. Introduction

Engine-driven nickel-titanium (NiTi) endodontic instruments with large tapers have become widely available. However, they have been identified as one of the potential etiologic factors of dentinal cracks [1–3], also referred to as microfractures or dentinal defects [4, 5]. Cracks may evolve to a root fracture and result in tooth loss [6].

Recently, microcomputed tomography (mCT) has been widely used to detect dentinal cracks in *ex vivo* and *in situ* studies [7–9]. The mCT offers some advantages when compared with the direct analysis of the root surfaces obtained by the cross-sectioning method because it allows a three-dimensional and nondestructive evaluation of specimens, and successive scans can be used after different treatment procedures. In addition, the sectioning approach could *per se* create new cracks [10]. On the other hand, mCT can lead to false-negative results because of several variables, such as the ones evaluated herein.

The mCT studies [7, 11–15] usually use scans taken before and after instrumentation to evaluate the formation of new dentinal cracks or the increase of preexisting defects. However, results from these studies are conflicting. While no new cracks were observed after root canal instrumentation in some studies [11–13], others reported the development of a significant number of defects [7, 14, 15]. Apart from each study's specificities that can partially explain these differences, including the instrument system, mCT parameters, and tooth type, problems related to the mCT accuracy should also be considered.

The level of accuracy of mCT for detecting cracks has not been appropriately defined so far. A recent study showed that the total number of cracks observed by stereomicroscopy was significantly higher than that by mCT [10]; however, no previous study correlated these two methods with a suitable reference pattern, such as scanning electron microscopy (SEM). Besides, the influence of the width of the crack in its detection has not been evaluated.

Therefore, the present study evaluated the accuracy of mCT to detect dentinal cracks when compared to SEM and operating microscopy. Different conditions of pixel size of the mCT (10 *μ*m or 17 *μ*m), sample moisture (dry or moist), and transillumination with the operating microscope (with or without) were evaluated. Moreover, the influence of the dentinal crack width on its detection was tested. The present study had the following null hypotheses: (1) there is no difference between mCT and the operating microscope in the detection of dentinal cracks; (2) the mCT resolutions of 10 *μ*m or 17 *μ*m do not affect crack detection; (3) the humidity conditions (dry or moist) do not interfere in crack detection by mCT; and (4) the crack width does not influence its detection by the different tested methods and conditions.

## 2. Materials and Methods

### 2.1. Specimen Selection, Canal Preparation, and Induction of Cracks

Approval for the study protocol was obtained from the Ethics Committee of the Iguaçu University, Nova Iguaçu, Brazil (approval number: 1696413). Twelve human mature mandibular incisors were obtained from the Institutional Human Tooth Bank. Reasons for extraction were not related to this study. After access cavity preparation, the teeth were decoronated at the level of the cementodentinal junction with the aid of a diamond disc (Brasseler, Savannah, GA, USA) under continuous water irrigation.

The root canals were explored with a size 10 K-type file (Maillefer; Dentsply, Ballaigues, Switzerland) until its tip was visible at the apical foramen under magnification (5x). This procedure was performed to measure the canal length, check for the presence of only one canal, and confirm the apical foramen was patent. The working length (WL) was established 1 mm short of the apical foramen. The canals were irrigated with 1 ml 2.5% sodium hypochlorite (NaOCl) and initially instrumented with a size 20 K-type file (Maillefer; Dentsply, Ballaigues, Switzerland) at the WL. After new irrigation, the canals were prepared with the Reciproc instrument R40 (40/0.06) (VDW, Munich, Germany). The instrument was moved in the apical direction using in-and-out pecking motions of 3 mm amplitude. The VDW Silver motor (VDW, Munich, Germany) was used to activate the instrument in the reciprocating mode. Three or four cycles were necessary until the instrument reached the WL. After each cycle, the instrument was removed and cleaned, the canals were irrigated with 1 ml 2.5% NaOCl, and the patency checked with a size 15 K-type file (Maillefer; Dentsply, Ballaigues, Switzerland). The irrigant was delivered by a 30-G NaviTip needle placed 3 mm short of the WL. After preparation, the smear layer was removed by rinsing the canals with 2 ml 17% ethylenediamine tetraacetic acid (EDTA) for 3 minutes, followed by 2 ml 2.5% NaOCl.

All teeth were sectioned 5 and 10 mm from the apex, using a diamond disc (Brasseler; Savannah, USA) under continuous water refrigeration, generating 3 segments per root (coronal, middle, and apical). Only the middle and apical segments were included in the study, totalizing 24 samples. These fragments were analyzed for the presence of dentinal cracks, first by SEM (the reference pattern), second by mCT, and third by an operating microscope.

### 2.2. Scanning Electron Microscopy

The root segments were analyzed in SEM at 10.00 kV, different magnifications varying from 80 to 3000x, and low-vacuum (Inspect S-50; FEI Company, Hillsboro, OR, USA). The coronal transverse surface of each root segment was examined for the presence of cracks. When a crack was detected, 2 micrographs were taken, one from the crack (800-1600x) and the other from the whole transversal surface (30-50x). The width of the selected crack was measured at the central point.

In the specimens exhibiting multiple cracks, only one was selected for the comparative analysis between methods and its major width was measured in the SEM. The selected one was more than 400 *μ*m long; when more than one defect met this criterion, selection followed the order of preference: complete canal crack (extended from the canal space to the external root surface), incomplete canal crack (extended from the canal for a distance into dentin but ended short of the external root surface), intradentinal crack (confined to dentin), and external crack (extended from the external root surface for a distance into dentin without communicating with the canal) [16]. The specimens with no cracks as observed by SEM served as negative controls.

### 2.3. Microcomputed Tomography

After SEM analysis, the root segments were scanned in a mCT scanner (SkyScan 1273; Bruker-microCT, Kontich, Belgium) operating at 114 KV and 70 *μ*A. Scanning was performed by using a rotation step of 0.5°, 360° around the vertical axis, and a 1.0 mm thick aluminum filter. Scanning was performed at two pixel sizes (resolution): 10 *μ*m and 17 *μ*m, under dry and moist conditions (groups: 10dry, 10moist, 17dry, and 17moist). The first scans were of the dry specimens. The dry condition was obtained by maintaining the teeth in a dry environment for 14 days. Next, the fragments were immersed and maintained in distilled water for two hours and were scanned again in both resolutions. Overall, each fragment was scanned 4 times. The average scan time for each fragment was 50 minutes. The image of each specimen was reconstructed using NRecon v.7.1.0 software (SkyScan 1273; Bruker-microCT, Kontich, Belgium). Standardized parameters included beam hardening (50%), ring artifact correction of 5, and smoothing of 0. The acquisition approach produced 400–600 transverse cross-sections per fragment.

As the samples were scanned 4 times (10 *μ*m and 17 *μ*m, under dry and moist conditions), the mCT calibration and the setting of fiducial markers on images were required to guide the registration procedure. For that, the Surface Registration option from the 3D slicer v 4.10.2 software (available at http://www.slicer.org) was used for superimposition of the images. This tool provides the Fiducial Registration, which uses fixed pairs defined on a fixed sample (used as reference), corresponding to movable sample (target), orientating the registration process. These points act as fixed landmarks (fiducials) in both samples and stay unaltered during the scanning procedure. Four pairs of fiducials points were used in each registration phase, on the mesial, distal, buccal, and lingual edges of the tooth samples.

After the registration procedure, the cross-section images of each root segment (*n* = 24) were carefully inspected for the presence of cracks using the CTAn v.1.16.4.1 software (SkyScan 1273; Bruker-microCT, Kontich, Belgium). The mCT images were evaluated independently by two examiners, blinded to the SEM findings. The level of agreement between the examiners was very high (Cohen's kappa = 0.98). Discordant cases were decided by a third examiner, who is an endodontist too.

### 2.4. Operating Microscopy

The same root transverse surfaces evaluated in SEM and mCT were examined with an operating microscope (ZEISS Pico; Carl Zeiss AG, Oberkochen, Germany), varioskop 200-300 mm, under 23x magnification, 120.000 lux LED illumination. Micrographs were obtained from the transverse surfaces with Canon T7i, with and without transillumination (groups: OMTrans and OM). The transillumination was performed with LED light curing with a power of 1200 mW/cm^2^ (UltraLight III). All samples were examined with a standardized object-to-microscope distance of 27 cm. The images were carefully inspected for the presence and location of cracks by two experienced endodontists independently, previously calibrated and blinded to the SEM findings (Cohen's kappa = 1.0, 100% of agreement).

### 2.5. Correlative Analyses

Correlative analyses were performed to address 4 questions:

1. What is the effectiveness of both mCT and operating microscopy in detecting a crack revealed by SEM?
2. Could the two tested mCT resolutions affect the detection of cracks?
3. Could the humidity conditions affect the detection of cracks by mCT?
4. Can the crack width influence its detection?

The transverse surface of each specimen was assessed and diagnosed as having a crack or not [17]. Sensitivity, specificity, and accuracy were compared using correct and incorrect responses taking into account the reference pattern (SEM). In the present context, sensitivity is the proportion of the specimens containing a crack that were correctly diagnosed as having the crack (true positive rate). Specificity is the proportion of the specimens without crack that were correctly diagnosed as not having a crack (true negative rate). Accuracy is the proportion of the diagnoses that agreed with the known sample condition.

### 2.6. Statistical Analyses

Sensitivity, specificity, positive and negative predictive value, likelihood ratios, and accuracy were calculated using Microsoft Office Excel 2007 (Microsoft, Washington, DC). A ROC curve was created using the SPSS software (version 18.0; SPSS Inc., Chicago, IL).

The analysis of the influence of the crack width on its detection was carried out as follows. Because the width of the cracks varied from 1.9 *μ*m to 25.93 *μ*m, data were divided into two categories: ≤12.5 *μ*m and >12.5 *μ*m, and the influence of crack size on its detection was compared for all evaluation methods. The one-tailed Fisher's exact test was used to compare the data of each method with the reference pattern (SEM). The level of significance was set at *a* = 0.05 for all statistical tests.

## 3. Results

**Figure 1:**
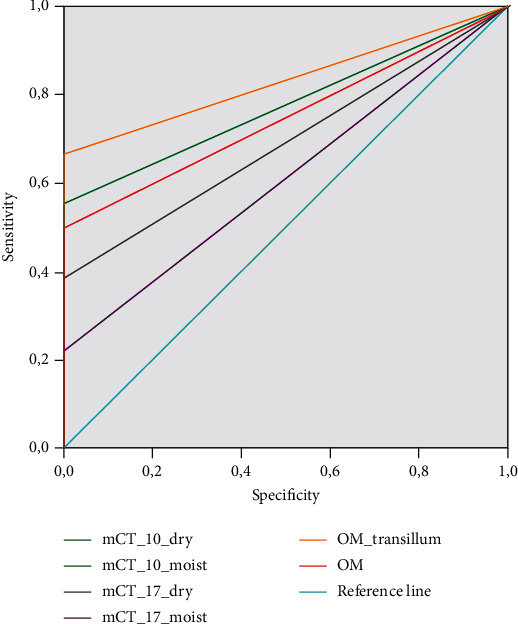
The ROC curve analysis for the tested methods and conditions. The larger the area under the ROC curve, the greater the accuracy.

**Figure 2:**
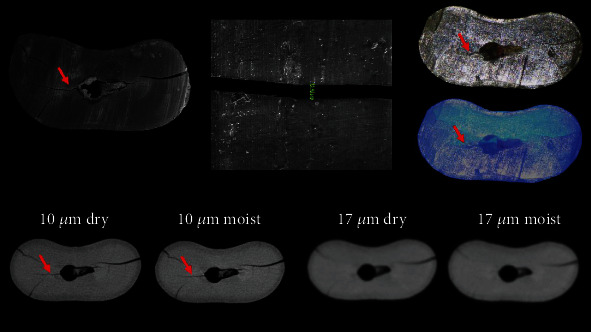
SEM, mCT, and OM of a same specimen showing the evaluated dentinal crack (red arrow) by the different tested methods. Note the crack disappeared with the worst resolution.

**Figure 3:**
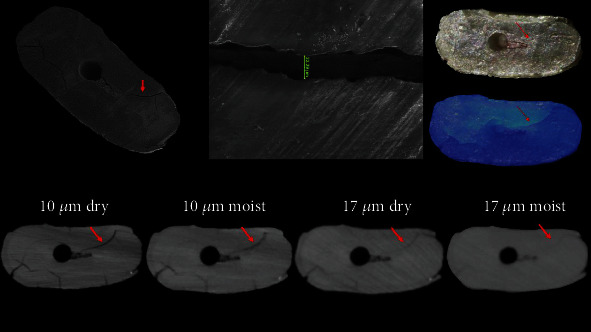
SEM, mCT, and OM of the same specimen showing the evaluated dentinal crack (red arrow) by the different tested methods. Note the crack was detected in all conditions, but its definition was getting worse.

From a total of 24 samples, SEM analysis revealed the formation of cracks in 18 specimens. Six specimens remained intact, without any detectable crack, and served as a negative control. The mean width of the measured cracks was 13.24 *μ*m (median = 12.50 *μ*m). Sensitivity, specificity, predictive values, likelihood ratios, and accuracy of mCT and operating microscope are shown in Table 1. Accuracy was moderate for the 10dry, 10moist, and OMTrans groups, poor for OM and very poor for 17dry and 17moist. The area under the ROC curve, which is another indicator of accuracy, was 0.83, 0.78, 0.78, 0.75, 0.69, and 0.61 for OMTrans, OM, 10dry, 10moist, 17dry, and 17moist, respectively (Figures 1–3). The larger the area under the ROC curve, the greater the accuracy. Some cracks visualized in SEM were not detected by mCT and an operating microscope (Table 1).

The thickness of the dentin crack significantly influenced its detection by mCT using the resolution of 10 *μ*m in both dry and wet conditions (*P* = .002), 17 *μ*m in the dry condition (*P* = .002), and by the operating microscope using transillumination (*P* = .009). Many cracks were not detected on the 17moist group. Virtually, all cracks thinner than 10 *μ*m passed unnoticed by all the methods used (Table 2).

## 4. Discussion

A valid reference pattern is required to validate the accuracy of any diagnostic method because it is essential to know the true state of the examined specimen [18]. Despite many published studies using mCT to evaluate the presence of cracks, the diagnostic accuracy of this method has not yet been determined. The operating microscope was also evaluated instead of a bench stereomicroscope because it can be a valuable diagnostic tool to detect dentinal cracks in the clinical setting. The detection methods were compared in the 18 samples with microcracks identified by SEM; this sample size was larger than a previous study [10] that showed that 10 samples are sufficient to compare the crack detection by mCT or cross-sectioning followed by stereomicroscopy.

Although this study used SEM, with low vacuum, which does not require sample preparation, it is possible that some cracks have been caused or increased during SEM procedures. However, because the purpose of the present study was to compare the process detection methods and taking into account that both mCT and operating microscope analyses were performed after SEM, this influence is not expected to be much of a concern. Additionally, the present study did not evaluate the cause of the crack but its presence.

There are some positive aspects related to the use of mCT for the detection of dentinal cracks. Unlike microscopic analysis, mCT permits a three-dimensional and nondestructive evaluation of specimens that can be used before and after root canal preparation procedures. In this way, preoperative cracks can be detected, and the specimens serve as their own control. Results of recent mCT studies revealed that cracks observed in postoperative images were already present in the preoperative period, denying a cause-and-effect relationship between the formation of dentinal cracks and root canal instrumentation [8, 12, 19]. On the other hand, other studies observed crack formation after preparation with different systems, also using the preoperative images as controls [7, 14, 15]. Inconsistencies between studies may be partly explained by the present findings, which showed that some dentinal cracks visualized by SEM could not be detected by mCT.

In the present study, all null hypotheses were rejected. Based on the mCT conditions adopted in the present study, the accuracy of mCT was considered moderate only with a high resolution of 10 *μ*m, independently of the moisture conditions. On the other hand, the accuracy was unacceptable at 17 *μ*m resolution in both moisture conditions. These findings showed that the accuracy of mCT for crack detection can not be satisfactory depending of the resolution and sample conditions. While 10 *μ*m was chosen because it is a very high-quality resolution, possible for the majority of mCT devices using tooth samples, 17 *μ*m was used because it is the mean value adopted by numerous previous studies [7, 9, 20], and the results may vary according to the equipment and the parameters applied to obtain mCT images. One should be aware that using a smaller resolution increases the sensitivity of mCT and further studies should evaluate if this increases the ability to detect microcracks.

One of the possible explanations for these results is related to the size of the crack. Our findings demonstrated that the width of the crack influenced its detection by mCT in groups 10dry, 10moist, and 17dry. As anticipated, no crack thinner than 10 *μ*m was detected by mCT with 10 *μ*m resolution; this occurred independently of the moisture conditions. Cracks were not detected by mCT or the operating microscope in any of the specimens with intact surfaces as revealed by SEM (negative controls), independently of the condition (no false positives).

A few studies have reported on what moisture conditions the mCT scanning was conducted [8, 9], while others have not [7, 12, 19]. This information is relevant because it has been speculated that the specimen storage conditions may affect the detection of dentinal cracks [9, 21]. The present study found that, when the resolution was optimized, the moisture conditions did not affect crack detection by mCT. This result contradicts a previous study [9]. There is no apparent explanation for this difference, but some points should be observed. One is that both studies agree that the crack visibility is compromised by humidity, although this has not impaired detection of the crack in the present study. The second point is the fact that only 5 samples with cracks were evaluated in the previous study [9], which is a small sample size. The final point is that the width of the crack can influence its detection, and this factor was not considered in the previous study [9].

The acquisition parameters may significantly affect not only the quantitative results but also the qualitative results of mCT analysis, especially the pixel size [22]. An increase in the pixel size provides relatively blurred images, which can compromise the crack detection, and lead to false-negative results, as observed in the present study.

The accuracy of OMTrans was slightly higher than that of mCT in optimized resolution. Transillumination improved the accuracy of the operating microscope, which was expected [23]. Some critical limitation of the operating microscope is the impossibility to evaluate cracks developing on the longitudinal axis, the inability to evaluate samples before and after the procedures, and the fact that it evaluates only the surface (then, the crack can only be detected if it is on the cut surface). For longitudinal cracks, even mCT studies analyzing individual cross-sectional slices have a problem related to the fact that slices in sequence of the same specimen can be affected by crack traveling among different horizontal sections [15].

## 5. Conclusions

The accuracy of mCT in detecting cracks was moderate in optimized resolution, independently of the moisture conditions, but lower than the operating microscope with transillumination. Considering the limitations of the present study, it was observed that the dentinal defect size can significantly influence its detection by mCT and an operating microscope. The accuracy of mCT was very low when a resolution of 17 *μ*m was used.

## Figures and Tables

**Table 1:** Prevalence of cracks and accuracy data for all evaluation methods and conditions.

Approaches	Sample size (*n*)	Prevalence (*n*)	Sensitivity (%)	Specificity (%)	PPV (%)	NPV (%)	LR+	LR-	Accuracy (%)
SEM	24	18	n/a	n/a	n/a	n/a	n/a	n/a	n/a
mCT 10 *μ*m dry	24	11	61.11	100	100	46.15	—	0.39	70.83
mCT 10 *μ*m moist	24	11	61.11	100	100	46.15	—	0.39	70.83
mCT 17 *μ*m dry	24	7	38.89	100	100	35.29	—	0.61	54.17
mCT 17 *μ*m moist	24	4	22.22	100	100	30	—	0.78	41.67
OMTrans	24	12	66.67	100	100	50	—	0.33	75
OM	24	9	50	100	100	40	—	0.50	62.50

PPV: positive predictive value; NPV: negative predictive value; LR+: positive likelihood ratio; LR-: negative likelihood ratio.

**Table 2:** Number of dentinal defects determined by the different approach.

Size of dentinal crack (SEM)	SEM	10dry	10moist	17dry	17moist	OMTrans	OM
<10 *μ*m	5	0	0	0	0	1	1
10-16 *μ*m	8	6	6	2	1	6	4
>17 *μ*m	5	5	5	5	3	5	4
Total	18	11	11	7	4	12	9

SEM: scanning electron microscope; 10dry: mCT with 10 *μ*m of resolution in dry condition; 10moist: mCT with 10 *μ*m of resolution in moist condition; 17dry: mCT with 17 *μ*m of resolution in dry condition; 17moist: mCT with 17 *μ*m of resolution in moist condition; OMTrans: op.

## Data Availability

All data are presented in the manuscript and can be available for all readers.
